# Next-Generation Human Cerebral Organoids as Powerful Tools To Advance NeuroHIV Research

**DOI:** 10.1128/mBio.00680-21

**Published:** 2021-07-13

**Authors:** Thomas A. Premeaux, Sonia Mediouni, Ana Leda, Robert L. Furler, Susana T. Valente, Howard A. Fine, Douglas F. Nixon, Lishomwa C. Ndhlovu

**Affiliations:** a Division of Infectious Diseases, Department of Medicine, Weill Cornell Medicine, New York, New York, USA; b Department of Immunology and Microbiology, The Scripps Research Institute, Jupiter, Florida, USA; c Meyer Cancer Center, Division of Neuro-Oncology, Department of Neurology, New York-Presbyterian Hospital/Weill Cornell Medicine, New York, New York, USA; Temple University; Albert Einstein College of Medicine

**Keywords:** cerebral organoids, HIV reservoirs, HIV cure, neuropathology, cure, HIV, reservoirs

## Abstract

Long-term effective use of antiretroviral therapy (ART) among people with HIV (PWH) has significantly reduced the burden of disease, yet a cure for HIV has not been universally achieved, likely due to the persistence of an HIV reservoir. The central nervous system (CNS) is an understudied HIV sanctuary. Importantly, due to viral persistence in the brain, cognitive disturbances persist to various degrees at high rates in PWH despite suppressive ART. Given the complexity and accessibility of the CNS compartment and that it is a physiologically and anatomically unique immune site, human studies to reveal molecular mechanisms of viral entry, reservoir establishment, and the cellular and structural interactions leading to viral persistence and brain injury to advance a cure and either prevent or limit cognitive impairments in PWH remain challenging. Recent advances in human brain organoids show that they can mimic the intercellular dynamics of the human brain and may recapitulate many of the events involved in HIV infection of the brain (neuroHIV). Human brain organoids can be produced, spontaneously or with addition of growth factors and at immature or mature states, and have become stronger models to study neurovirulent viral infections of the CNS. While organoids provide opportunities to study neuroHIV, obstacles such as the need to incorporate microglia need to be overcome to fully utilize this model. Here, we review the current achievements in brain organoid biology and their relevance to neuroHIV research efforts.

## INTRODUCTION

Although antiretroviral therapy (ART) has been effective in suppressing viremia and decreasing mortality and morbidity, human immunodeficiency virus (HIV) remains a chronic disease requiring lifelong treatment due to the persistence of viral reservoirs. Thus, a cure for HIV remains a crucial public health need. Cessation of ART in people with HIV (PWH) ultimately results in the resurgence of virus in the periphery (1, 2), with evidence showing anatomical compartments, such as lymphoid organs, gut-associated lymphoid tissue, and the brain, being likely sources of viral recrudescence (3–6). These tissue sites of viral persistence have hindered current efforts in the eradication of HIV. Evidence suggest that long-lasting viral reservoirs are established in the central nervous system (CNS), likely due to poor drug penetration and reduced immune surveillance, among other factors (7, 8). The CNS compartment should thus be taken into consideration in designing and implementing effective HIV curative strategies permitting ART-free remission. Furthermore, PWH continue to experience high rates (>50%) of cognitive abnormalities even when HIV replication is suppressed by ART (9–14), despite variations in prevalence by cohort and means of cognitive assessments (15–18). These neurocognitive deficits interfere with psychomotor speed and coordination and diminish memory and executive functions, reducing quality of life (19, 20), and current ART regimens have had variable impacts in reducing these deficits (21).

HIV penetration into the CNS is thought to occur early during acute infection, likely through the trafficking of infected lymphocytes and/or myeloid cells during heightened systemic immune activation across the blood-brain barrier (BBB) (22–30). Once within the brain parenchyma, HIV can infect and integrate into the genome of permissive resident cells, such as microglia and perivascular macrophages (19). Evidence exists that microglial cells and perivascular macrophages constitute the major cellular HIV brain reservoirs, with astrocytes being potentially another cellular reservoir (31–37). HIV RNA and associated viral protein expression in human brain tissues has been reported (38–40), with recent studies showing macrophage-tropic HIV type 1 (HIV-1) replication in myeloid cells in the CNS of individuals with HIV-associated dementia (41). In humans, these studies have mostly been limited to the examination of postmortem brain tissue. Addressing viral persistence and neuropathological mechanisms in the human brain remains a challenge, supporting alternate model systems to advance research on HIV infection of the brain (neuroHIV). Large- and small-animal models have been widely used to address these issues (42, 43). Evidence from simian immunodeficiency virus (SIV) nonhuman primate (NHP) studies have shown that CNS infection occurs within days of exposure, viral DNA can be detected in the brain during plasma viral suppression, and replication-competent virus resides in both perivascular macrophages and microglia (44–46). NHP models can simulate HIV disease in the CNS better than *in vitro* assays; however, these costly and time-consuming experiments still do not faithfully predict treatment outcomes and have not fully shed light on the underlying mechanisms of HIV-induced CNS disease (47–49). Genetically modified mouse models have been a useful tool for the understanding of molecular and cellular mechanisms and development of therapeutics for neuropathological infections (50). More recent studies using HIV-infected humanized mice and conventional mice infected with chimeric HIV (EcoHIV) have furthered the understanding of viral reservoirs in the brain and the impact of HIV on cognition (51–53). While animal models provide insight into retroviral dynamics within a host, there are still limitations in recapitulating the pathology, transcriptional phenotypes, and functionality to in comparison to that of human CNS infection.

Evaluations of mechanistic outcomes of HIV and human-specific CNS cell interactions have been predominately relegated to two-dimensional (2D) tissue culture models. Immortalized microglial cell lines, such as HMC3 and C20, have been heavily used as HIV infection models (48, 54), while generated latently HIV-infected microglia models, immortalized human microglia carrying a single round HIV construct, have elucidated potential mechanisms for HIV reactivation from latency (48, 55, 56). While peripheral blood monocyte-derived microglia (MMG) have also been used and shown to be productively infected with HIV infection (47), recent technological advancements in human induced pluripotent stem cells (hiPSCs) allowed the generation of microglia that more accurately represent the phenotype of primary microglia and are permissive to HIV (57). Human astrocyte cultures have shown that cellular entry of HIV is not through the classic CD4/gp120 fusion but rather by cell-to-cell contact with infected cells, the engulfment of infected cells, or the internalization of HIV through endocytosis (58–60). Several studies indicate that a small fraction of astrocytes can be transiently productive and latently infected by HIV *in vitro* (36, 61). However, the detection of virions and components within astrocytes from the engulfment of entire infected cells and through the endocytic cycle of HIV internalization and release can be mistaken for productive infection and integration (60).

More recent coculture 2D models that incorporate multiple CNS resident cells have demonstrated the importance of intercellular dynamics in regulating neuroinflammatory responses and HIV kinetics. Healthy neurons have been shown to mediate HIV transcriptional silencing in microglia when cocultured (49). Tricultures, containing human iPSC-derived neurons, astrocytes, and microglia, demonstrate that microglia are the predominate cells that are productively infected as well as that cellular cross talk elicits distinct microglial functional and proinflammatory signatures. Furthermore, infected and HIV-exposed microglia, in these tricultures, show impairments in cell cycle regulation, phagocytoses of synapses, and DNA repair mechanisms (62). While essential for neuroHIV research, these 2D cultures lack the 3D complexity and the complete cellular composition of the brain and, thus, do not represent the actual cellular environments or fully recapitulate human disease or treatment effectiveness.

The reprogrammable nature of human pluripotent stem cells (hPSCs) and innovations in stem cell technology have facilitated the generation of organoid technologies, 3D culture systems that undergo some level of self-organization and resemble *in vivo* organs (63–65). Brain organoids offer the possibility to investigate cellular development and intercellular interactions within a 3D human brain microenvironment. Brain organoids, which maintain the genotype of the original cell or tissue source, are more heterogeneous and complex than 2D models and have provided useful insights into human brain development and a variety of neurological disorders and neurotropic infections (64, 66, 67). Given the limitations of current *in vitro* models, the accessibility of primary human CNS cells, the restriction of infecting rodent CNS models only with the substantial manipulation of the host or HIV, and the expense and expertise required to examine nonhuman primates, many HIV investigators are turning to human brain organoids in hopes of having an affordable, physiological, and reproducible model to study HIV disease mechanisms and predictors of treatment efficacy in the CNS.

## ADVANCES IN HUMAN BRAIN ORGANOID TECHNOLOGY

### Brain organoid development, advantages, and limitations.

**FIG 1:**
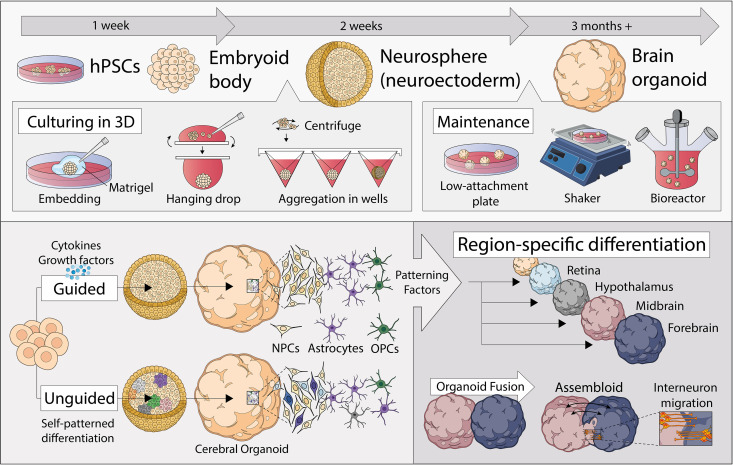
Advances in human brain organoid development, including three-dimensional brain organoids from human pluripotent stem cells (hPSCs) and various means of their production and maintenance. Organoids can be generated under undirected conditions through self-patterned differentiation or directed methods using patterning factors to produce brain-specific regions, which can be subsequently fused to form “assembloids.”

hPSCs, including human embryonic stem cells (hESCs) and hiPSCs, are able to differentiate into any cell or tissue type, under specific cues and favorable conditions (68). hPSCs are initially differentiated into embryoid bodies, further toward the neuroectodermal lineage as simple clusters/spheroids, and then to a more complex organization resembling the intricacy of the human brain (Fig. 1). Typical methods of achieving a three-dimensional structure by culturing cells under low-adhesion conditions include the hanging-drop method, cell aggregation in U- or V-bottom well plates, and embedding cells in extracellular matrices (64, 69, 70). Continued growth and maintenance of brain organoids generated can be done in low-attachment plates spun in a bioreactor (a culture system that allows nutrients to be supplied while cells are agitated) or shaken in tissue culture plates. The generation of brain organoids, using these methods, can be under undirected (or unguided) (64, 71) or directed (or guided) conditions (72–74). Undirected organoids are generated in the absence of inductive cues and rely on the intrinsic signaling and self-organization capacities of hPSCs, allowing them to stochastically give rise to cells resembling those found in multiple brain regions, ranging from the retina to hindbrain (63, 75, 76). Alternatively, specific combinations and timing of exogenously applied signaling molecules and growth factors (i.e., Hedgehog signaling) can “direct” brain organoid development to generate specific regions of the brain (64, 72, 73, 77–85). Unguided organoids are more suitable for exploring cell type diversity during whole-brain development, while brain region-specific organoids better recapitulate the brain cytoarchitecture with less heterogeneity that allows for better investigation between specific brain regions with more consistent molecular and functional characterization (63, 75, 76).

Brain organoids recapitulate several key features of *in vivo* organogenesis, which allows the interrogation of a working structural human brain *in vitro* (64). Brain organoids also possess neurons that are functionally capable of electrical excitation and exhibit neuronal transcriptomes that parallel those found in the human brain (86). However, the cellular diversity and structural complexity of brain organoids highly depend on its state of maturity or length of culturing. At the morphological level, immature brain organoids (∼3 months of culture) present a rosette-like structure, containing neuroepithelial stem cells and ventricular radial glial cells that divide at the apical surface and form a ventricular-like zone (65). These present, however, less cell type diversity than those that are more mature. Mature organoids (≥6 months of culture), however, have multiple progenitor zones, including a ventricular-like zone and an outer subventricular-like zone formed by outer radial glial cells that express specific cortical layer and glial cell markers (65, 73). These mature brain organoids also resemble human cortical development at the gene expression level and allow in-depth analysis of neural networks, cell behavior, drug screening, and disease modeling (87). Single-cell mRNA analysis of hPSC-derived brain organoids revealed a multitude of distinct cell populations in mature organoids, including astrocytes, neuroepithelial progenitors, oligodendrocyte precursor cells, neuronal-lineage cells, cells enriched for forebrain markers, and cells expressing retina-specific genes and displayed an enrichment of genes associated with neuronal and glial maturation (71). Although the majority of brain organoid generation protocols aim at modeling cortical development, upon differing culture “guided” conditions they can also form a variety of brain regions, including the cerebrum, midbrain, retina, choroid plexus, and hypothalamus, that all mimic the signaling patterns of their respective brain structures (64, 72, 73, 77–84). While brain region composition varies in organoids from different iPSC lines, regional human-specific gene expression patterns remain largely reproducible across individuals (88). Therefore, brain organoids recapitulate the characteristics of the human brain not only at the cellular level but also in the architecture and developmental trajectories (63).

The 3-dimensionality of brain organoids offer several advantages over 2-dimensional models. Brain organoids are more physiologically relevant, as they can more accurately recapitulate the spatiotemporal organization of the human brain, grow in a more realistic way like cells and organs *in vivo*, and reproduce key aspects of human brain function. Cells within organoids also display their natural cell shape, exhibit gene and protein expression levels resembling levels observed in cells *in vivo*, form multiple layers, have realistic proliferation rates, and can retain homeostasis for longer periods (89–91). Brain organoids can model how different cell types interact and respond to flow differentiation and metabolic adaptation, allow efficient cell-to-cell communication (as cell junctions are highly abundant), and more accurately represent responses to mechanical stimuli of cells (89–92). Brain organoids also provide an advantage over other organ-like 3D tissue culture systems, such as neurospheres, that are 3D aggregates of CNS cell types derived from neuronal progenitor cells without a cytoarchitecture (93) or “organ-on-a-chip” technology composed of neural cells artificially assembled in culture chambers and biomaterial scaffolds (94).

Despite the advantages of brain organoids as a model system to study brain pathology, gas and nutrient exchange within organoids, organoid heterogeneity between batches, recapitulating structural components of the human CNS, and increasing cellular diversity need to be addressed to advance the physiological relevance of these organoid models in neuroHIV pathology. To address nutrient and gas exchange, organoids can be grown in bioreactors, in orbital-shaking incubators, or at the liquid-air interface; however, apoptotic cell death within center regions of the organoid due to lack of oxygen with long-term culture could remain a problem (95, 96). Major consideration should be given to batch effect when choosing the ideal organoid generation method, as the stochastic nature of differentiation in unguided organoids coupled with the intrinsic differentiation propensities of individual hPSCs results in high interorganoid variability (71). Transcriptomics data show that bioreactors have helped decrease organoid heterogeneity between batches, as organoids grown in the same bioreactor tend to have more similar cell types, suggesting that organoids secrete signaling factors that influence sister organoids (71, 97). To improve structural organization, some groups have begun fusing individual region-specific organoids into larger “assembloids” that generate cortical interneurons to allow modeling interconnectivity between multiple brain regions (76). Furthermore, as brain organoids derive from a neural lineage cell population, they lack other CNS cells like microglia and vascular cells, which play essential roles in proper brain function. However, there have been recent exciting studies directed at the interplay of these CNS resident nonectodermal cell types in organoids (63, 75, 76), which allow a greater degree of cellular and network maturity.

### Cerebral organoids, a tool for the investigation of neurotropic viruses.

While human brain organoids have been essential in elucidating mechanisms of neurodevelopment and neurodegenerative disorders in the past decade (64, 98–100), they have also recently been used as viable alternatives to animal models to enhance investigation of neurotropic viruses, including the study of host-pathogen interactions and associated neuropathological outcomes. Brain organoids have been used to study neurotropic viruses associated with diseases of major public health concern worldwide in which the pathogenesis is still poorly understood, including Zika virus (ZIKV), herpes simplex virus (HSV), and human cytomegalovirus (HCMV), and in recent studies evaluating the ramifications of severe acute respiratory syndrome coronavirus 2 (SARS-CoV-2) infection on the CNS. While *in vitro* stem cell-based and *in vivo* animal models helped elucidate cell specificity and brain development with CNS viral infection (101–103), these can lack brain complexity and differ in viral entry receptors (104–106), which brain organoids can address.

ZIKV, declared a public health emergency in 2016, has been largely studied in brain organoids. The strong relationship between newborns with microcephaly and ZIKV infection propelled the use of iPSC-derived organoids, immature and many guided as forebrain region specific, to model the effect ZIKV has on the developing brain (107). Brain organoids revealed that ZIKV infection causes neuronal cell death at the early stages of brain development, the dysregulation of neurogenesis, and the premature differentiation of infected neural progenitor cells (73, 101, 108–111). Although no specific therapeutic antiviral has yet been developed, substantial knowledge on the effects of ZIKV infection on fetal brain development was gained, including that ZIKV-associated microcephaly could potentially be due to the abrogation of neurogenesis and cortical thinning from neural progenitor cell NPC depletion induced by ZIKV (73, 108, 110).

Neonatal infection with herpes simplex virus (HSV) occurs in 1 out of every 3,200 to 10,000 live births, resulting in high mortality or permanent neurological sequelae (112–114). The use of brain organoids has shown to be valuable in the attempt to elucidate the primary molecular mechanisms of HSV-1-driven neuropathology that previously were difficult to study. In immature brain organoids, HSV-1 efficiently infected the complex laminar structure, was transported from the periphery to the central layers, and was able to be effectively reactivated from latency (115). HSV-1 infection also impaired neurogenesis and dysregulated the cortical layer along with brain regionalization during the active period of proliferation of the neuroepithelium (15 days), while in the later stages of infection (45 days), neuronal differentiation was compromised, and numerous inflammatory factors were markedly released (116). Furthermore, much like ZIKV, HSV-1 impaired brain organoid development and induced distinct morphological changes (117).

HCMV neuropathogenesis is the most common cause of infectious-related birth defects. While animal models have elucidated many aspects of brain abnormalities and neuropathogenic mechanisms induced by CMV congenital infection, the main caveat lies in the strict host specificity of CMVs (118, 119). To illuminate human-specific effects of HCMV infection, iPSC-derived human brain organoids were infected with the widely used strain TB40/E, which resulted in reduced brain organoid growth, impaired formation of cortical layers, and abnormal calcium signaling and neural network activity (120–122). Additionally, the use of organoids to model CMV infection confirmed both epidermal growth factor receptor and platelet-derived growth factor receptor A in facilitating viral entry into human CNS resident cells, as well as the efficacy for the use of monoclonal antibody therapeutics in the human brain, specifically those that target the envelope pentamer glycoprotein of HCMV (120).

Individuals with severe coronavirus disease 2019 (COVID-19) have experienced severe neurological symptoms, such as encephalopathy, Guillain-Barré syndrome, and Miller Fisher syndrome (123–125), and more recently, iPSC-derived human brain organoids have been used to investigate the impact of SARS-CoV-2 infection on the CNS. Robust antiviral antibody titers in the cerebrospinal fluid (CSF) were observed in COVID-19 patients and human autopsy samples demonstrate the presence of SARS-CoV-2 RNA transcripts in brain tissues as well as viral proteins in olfactory bulb endothelial cells (126–128). Furthermore, autopsy data indicate that neurons are not directly infected, rather vascular and immune cells with indications of microglia and astrocyte activation (129, 130). To investigate the ramifications of COVID-19 in the human brain, mature iPSC-derived human cortical organoids were infected with SARS-CoV-2 (131–133). Brain organoid models demonstrated that infection was mediated through the ACE2 receptor, resulting in the active release of progeny virions. This contrasted with *in vivo* data, which showed entry into cortical neurons and neuronal progenitor cells (132, 133). Furthermore, cellular pathways related to cell division, organelle fission, and metabolic processes were upregulated in neurons, with SARS‐CoV‐2‐positive neurons exhibiting an altered distribution of Tau from axons to soma, hyperphosphorylation, and ultimately cell death within 2 days postinfection (131). Exposure at different developmental stages of the organoid showed a preferred tropism for mature neurons (131, 133); however, another study showed that the choroid plexuses are primarily infected, with only large amounts of virus leading to neuronal and glial infection subsequently (134).

Contrasting results in the SARS-CoV-2-infected brain organoid illustrates the need to improve methodologies to address accuracy, robustness, and reproducibility between laboratories. Furthermore, the lack of CNS resident cells not belonging to the neural lineage, such as vascular cells and microglia, could be a major impediment in accurately assessing the impact of this neuroviral infection. For example, in SARS-CoV-2 infection, endothelial cells are suggested to be important in the progression of COVID-19 (135); thus, brain organoids without a vascular system could be missing key elements in COVID-19-related CNS complications. While current human brain organoids have facilitated breakthroughs in neurotropic viral infections, their continuous development is still needed to allow standardization and their use in high-throughput screenings for compound discovery as well as to address HIV-specific outcomes.

## OPPORTUNITIES TO STUDY HIV IN HUMAN BRAIN ORGANOIDS

Persistent or latent HIV infection of specific brain regions may induce chronic inflammatory changes which play pivotal roles in the pathophysiology of cognitive disorders in PWH on ART. While it is inconclusive that HIV directly infects neurons (136), the presence of HIV RNA transcripts, proteins (137), and proviral DNA (138) has been detected in neurons. Astrocytes, the most abundant cell type in the CNS (20 to 55%), also play a major role in HIV CNS disease, as they are shown to be permissive to HIV, although with limited replication, and produce robust expression of viral proteins (60). Many HIV proteins are known to activate CNS resident macrophages and glial cells and induce them to produce factors that mediate neuronal injury and apoptosis, such as proinflammatory cytokines and reactive oxygen species. As functional neurons and astrocytes are present in cerebral organoids, these processes could be easily modeled in a 3D environment with intercellular networks. However, evidence shows that HIV remains persistent in myeloid and microglial cells, and the identification and quantification of these reservoirs in virally suppressed patients present a major roadblock to HIV cure research (139–141).

Microglia, the CNS resident tissue macrophages, can comprise up to 17% of all cells in the adult brain depending on region and play multiple roles in health and disease. Microglia shape neuronal plasticity through pruning and stripping synapses and participating in bidirectional signaling with closely intertwined neurons to promote efficient neuronal circuits (142). Microglia can regulate the number and diversity of neurons by triggering apoptosis and phagocytizing dead neurons (143, 144). Microglia also secrete a wide array of factors that impact neuronal function, including those that promote neurogenesis (145, 146). Beyond microglia serving as a viral reservoir, they are shown to exhibit excessive or unchecked activation and an altered proteome with HIV infection that contributes to associated cognitive impairments (147). Therefore, to fully recapitulate events of HIV infection and persistence in the human brain, microglia incorporation into organoids would be highly beneficial to study neuroHIV and to develop comprehensive strategies to prevent or treat CNS complications in HIV.

A recent study showed microglia-like cells can innately develop within iPSC-derived cerebral organoids and exhibit characteristic morphology and gene and protein expression, as well as function like adult human microglia. However, these innate “microglia” are not shown to occur in high numbers (148). Several studies recently reported the incorporation of microglia and myeloid cells as immune mediators in human brain organoid models; however, the source of these cells is crucial in mimicking the phenotype of microglia and their infectibility by HIV-1 (57). The sources of microglia and myeloid cells in published models include primary cells, immortalized cell lines, and stem cell-derived microglia. Incorporation of primary microglia is ideal for modeling HIV-1 disease in the CNS, as these cells are readily infected by HIV-1 and retain phenotypic markers (i.e., IBA-1, high CX3CR1, P2RY12, and TMEM119) (149, 150). However, despite their physiological relevance, primary microglia must be harvested from postmortem biopsy specimens or derived from fetal brain tissue, which limits their availability. In addition, since they are terminally differentiated, they do not proliferate substantially and have a limited life span in culture, making them less useful for HIV studies using organoid models.

To overcome this barrier, groups have added immortalized microglial cell lines or iPSC-derived microglia to organoids to mimic primary microglia in the CNS. A recent study utilizing the HMC3 microglial cell line showed that both uninfected and HIV-infected microglial cells migrated into NPC-derived spheroids, resulting in productive viral infection, and exhibited augmented inflammatory responses (151). However, commonly used microglia cell lines, such as HMC3 and C20, are reported to express low levels of CD4, and their infection is limited primarily to pseudotyped virus (48, 57). They are also limited by their relatively poor expression of genes relevant to HIV replication (i.e., SAMHD1, BST2/tetherin, and APOBEC3G genes) and retain little, if any, of the surface markers displayed on human microglia (CD11b, CD45, and SIGLEC1) (54, 57). Distinct transcriptional profiles are also observed between human immortalized microglial cell lines and primary microglia and MMG, making these cell lines less relevant for studying HIV (57). Both MMG and monocyte-derived macrophages (MDMs) express CD4 and can be productively infected with R5-tropic HIV, providing justification for their use in brain organoid models. However, infection of MMG and MDMs results in lower viral production and apoptosis than infection of primary microglia, which may be due to differing culture conditions and viruses used for infection (57, 152). While viral production occurs for a longer period in primary cells, these terminally differentiated cells exhibit low levels of cell proliferation and have a limited life span in culture.

Recently developed iPSC-derived microglia retain CD4, support ongoing HIV replication, and have been incorporated into brain organoid models (57, 140, 153–155). Several studies show that primary and iPSC-derived microglia dramatically alter their gene expression and phenotype when cultured *in vitro* under different conditions (140, 155–160). However, a recent study shows that neurons and astrocytes drive specific microglial identity and regulate their phagocytic function and inflammatory responses *in vitro* (161), indicating that signaling from other CNS resident cells may be required for these microglia to mimic *in vivo* counterparts. In a study in which iPSC-derived microglia were aggregated with predifferentiated neural cultures at a postmitotic and gliogenesis stage, NPC-derived spheroids were formed with embedded microglia that were highly branched (140). Similarly, migration and ramification of iPSC-derived microglia in cortical organoids were observed when these were cultured for more than 100 days (153), and iPSC-derived microglia have been incorporated into a 2-month-old cerebral organoid model of Alzheimer’s disease (154). Additionally, iPSC-derived microglia that were incorporated into cerebral organoids resembled microglia *in vivo* and responded to injury stimulus by migrating to the injury site and becoming activated (155).

**FIG 2:**
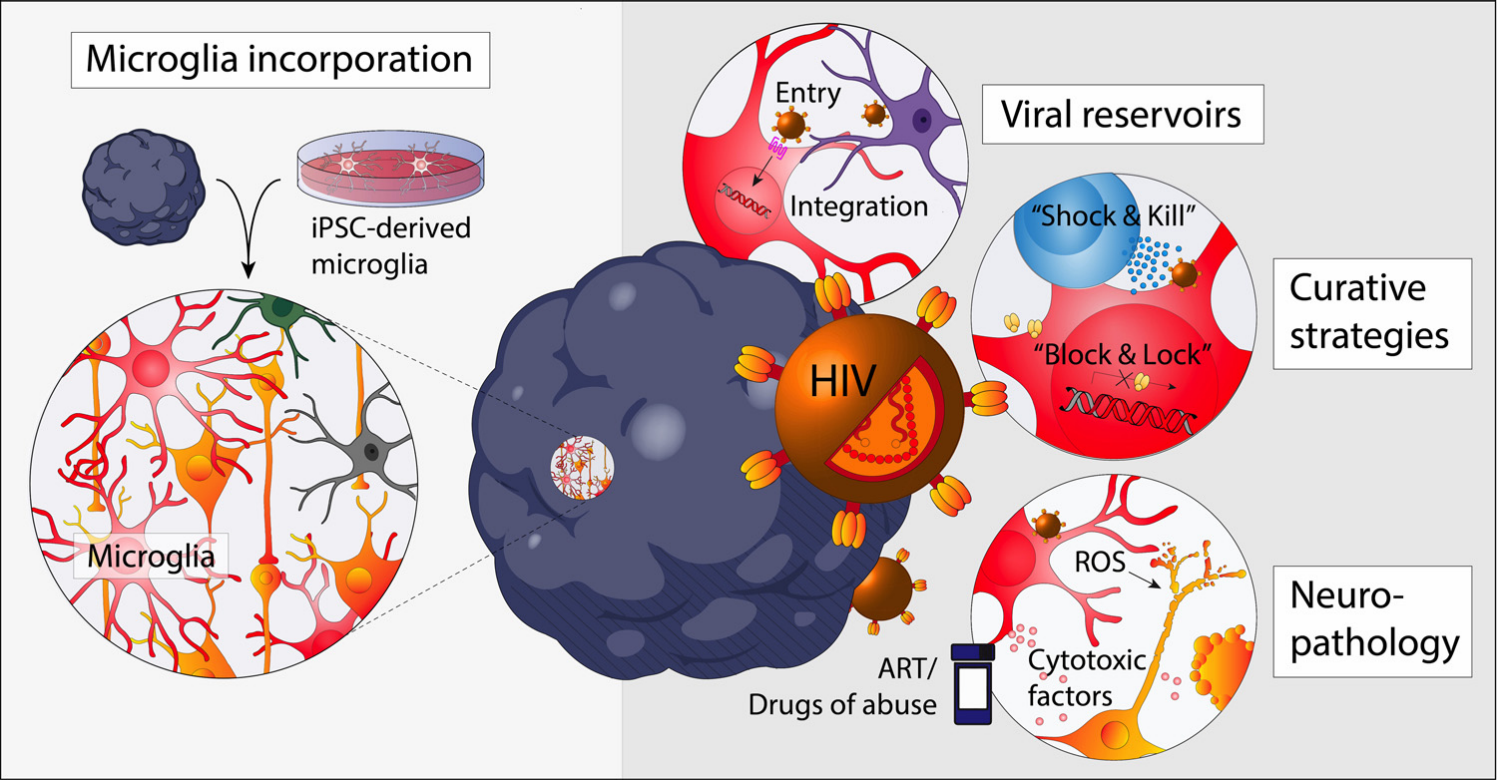
Opportunities to study neuroHIV using brain organoids. Brain organoids with the incorporation of microglia allow the study of neuroHIV, including cellular reservoirs, curative strategies, and neuropathological outcomes due to HIV infection, microglial activation (reactive oxygen species [ROS] and proinflammatory cytokine secretion), and toxicities from antiretroviral therapy or drugs of abuse.

Although brain organoid usage is not yet commonplace in the HIV research field, recent advances in organoid development and composition open the opportunity to model the impact of various aspects of neuroHIV, including viral reservoir establishment, curative strategies, and HIV-induced neuroinflammation and neuropathology, as well as the effects of antiretroviral toxicity and additive pathologies following substance abuse (Fig. 2). Advances in brain organoid have led to efforts to investigate HIV, but many ongoing studies are limited by the lack of microglia in these models. Attempts to overcome this issue are currently being made, specifically by coculturing two doxycycline-inducible iPSC sublines that can differentiate into either microglia or excitatory neurons, incorporating iPSC-derived microglia into already formed brain organoids, or the simple coculture of iPSC-derived microglia and brain organoids.

Reservoir studies are attempting to identify key intrinsic signals mediating viral silencing and reactivation. Brain organoids could be particularly efficacious in elucidating the efficacy of agents for HIV curative measures that either silence HIV transcription (the “block-and-lock” approach) or promote latency reversal from cellular reservoirs for the subsequent killing by effector immune cells (the “shock-and-kill” approach) (162–164). Autologous immune cells, including HIV-specific cytotoxic T lymphocytes (CTLs) and natural killer cells, can be added to autologous induced progenitor cell-derived CNS organoids to evaluate effectiveness of various cell therapy cure strategies in eliminating productively and latently HIV-infected cells, a method found effective in NHP models and lymph node organoid cultures (165). CTL-mediated “killing” methods are often paired with latency-activating agents, such as histone deacetylase inhibitors and Toll-like receptor agonists, that “shock” these latently infected cells to express HIV (166, 167). Studies under way are also evaluating the alternative block-and-lock approach to silence HIV long term, specifically using CRISPR interference by promoting H3K9me3 and DNA methylation or using didehydro-cortistatin A (dCA), an inhibitor of the HIV transactivator of transcription (Tat) protein (168–171). The use of brain organoids also opens avenues for testing other potential curative approaches for the CNS compartment, including using CRISPR to mutate or excise the HIV-1 genome, neutralizing the activity of HIV-1 resistant strains using broadly neutralizing antibodies, and ART intensification (172–175).

Importantly, microglial activation and neuronal damage associated with HIV infection are being investigated, which both have been linked to poor cognitive performance in ART-suppressed PWH (176–178). Current research is aimed at determining how HIV infection alters microglia-intrinsic pathways, neurodegeneration induced by HIV-infected microglia, along with the effects on organoid structure, neural network health, and signaling. Studies are under way to investigate how individual viral proteins cause neurological damage, namely, Tat and gp120, which are shown to be highly toxic to neurons and glia cells (179, 180). Brain organoid studies may also have translational potential, through corroborating with gene expression findings for microglia and neurons obtained from postmortem tissues of donors that exhibited HIV-associated cognitive impairment. This opens research avenues into novel therapies, namely, pharmacological agents against microglial receptors and/or mediators of inflammation, to potentially alleviate neuropathological outcomes and/or to delay the development of cognitive complications.

Substance abuse and addiction are highly prevalent in PWH, and many drugs of abuse (DOA), including opioids, methamphetamine (meth), and cocaine, are thought to exacerbate the effects of HIV-associated cognitive impairment (181). Glial cells are thought to be an important cellular site for drug-HIV-1 interactions (182). Morphine is shown to increase HIV replication in infected macrophages and microglia *in vitro* (183–185). Meth potentiates gp120-induced microglial neurotoxic activity *in vitro* (186) and increases the proportion of infected microglia/macrophages in NHPs (187). BBB *in vitro* models have shown that DOA impact the integrity of the BBB and promote the transmigration of monocytes across this layer (188), factors that are prevalent with HIV infection and that contribute to neuropathogenesis (189, 190). The adverse effects of several DOA alone have already been investigated with brain organoids, including methadone and cocaine (189, 191). Methadone has been shown to influence neuronal growth and function, including suppressing neural network activity and synaptic transmission (192), while cocaine limits neuronal differentiation and neural tissue development (193). However, the effect of HIV and drug coexposure on the CNS is not fully understood, and brain organoids would provide an efficient model of these processes. Current proposed studies to investigate these processes in brain organoids are now under way, aiming to unravel the impact of DOA on neuron-microglia signaling and microglial inflammatory responses. These novel approaches using organoids overall will likely result in a better understanding of the cell and molecular mechanisms underlying the additive effects of HIV and DOA on the brain.

As organoid models containing microglial and myeloid cells advance to study HIV disease within the CNS, parallel attention needs to be placed on methods of assessing HIV-induced neuropathology. Current qualitative and quantitative assessments of CNS pathology in organoid models evaluate cell subset viability and their infectibility, proinflammatory cytokine fluctuations, and synaptic integrity. In brain organoids, cytotoxicity can be measured by fluorescent dyes (i.e., calcein AM and ethidium homodimer) paired with microscopy or flow cytometry and specific cell subsets using subset-specific markers for neurons (βIII-tubulin and MAP2), astrocytes (GFAP), and microglia (IBA-1) (148, 194). Proinflammatory cytokines (i.e., tumor necrosis factor alpha [TNF-α] and interleukin 1β [IL-1β]) can be measured as surrogate markers of virus-induced neuroinflammation (195). The use of fluorescent or luciferase reporter viruses will help assess cell-specific infection by HIV, while viral antigen (p24) and viral RNA production could be used as readouts for productive HIV infection (196, 197). Furthermore, with the growing ability and sophistication in mapping intracellular neuronal-neuronal, glial-glial, and neuronal-glial electric signaling networks (i.e., optogenetics, multiphoton microscopy with genetic calcium transient reporters, and matrix electrical grids) (198–200), HIV disruption of normal cerebral physiology can be investigated by mapping “normal” electrical signaling in brain organoids with uninfected microglial/macrophages compared to that in individuals infected with HIV.

## FUTURE PERSPECTIVES

The research field of neuroHIV has made significant strides in elucidating the mechanism of viral persistence and the onset of CNS dysfunction through 2D *in vitro* models and animal surrogates; however, these have limitations related to their cost and to not fully recapitulating the complex and dynamic interactions of HIV and CNS resident cells. The development of brain organoid technology has facilitated the study of the neuropathogenicity of neurotropic viruses at different stages of brain maturity. The use of human brain organoids opens research avenues in investigating novel insights into the pathogenesis of HIV-associated cognitive impairment and neurotoxicity, as well as adding exciting findings on HIV-1 latency in the brain. While microglia are still lacking in most brain organoid models, their incorporation has been successful and significant efforts for their further incorporation are ongoing given their relevance in CNS HIV infection. Developing a model that incorporates microglia and/or perivascular macrophages is essential for neuroHIV research, as it would support robust HIV-1 infection, allow the testing of curative strategies, and facilitate the study of HIV-associated neuropathology. However, as the understanding of viral reservoir composition and activity in the CNS is still evolving, including the roles of both astrocytes and microglia, brain organoids could address the true nature of the cellular and frequency of latent HIV.

Recent brain organoid models developed to include a vascularized system addresses the size limitation of organoids but also allow investigation of endothelial dysfunction and BBB integrity. HIV, viral proteins, and inflammatory mediators induce structural and functional damage to the BBB and alter its permeability (201–203). This increased BBB permeability occurs early in acute HIV infection and can persist in PWH up to a year after ART initiation (191, 204–206). While *in vitro* BBB models have been essential in investigating HIV-associated endothelial dysfunction and BBB disruption, HIV research would benefit from insight into the interplay of neural and endothelial cells and the effects of infection on vascular integrity in a 3D system. Recent models of transplantation of human brain organoids into mouse brains show that the vascularization of brain organoids is an important aspect to facilitate progressive neuronal differentiation and maturation and gliogenesis (207, 208). The incorporation of human E26 transformation-specific variant 2-expressing cells or homologous iPSC-derived human endothelial cells show that organoids become more complex with prolonged culture to form a vascular-like network with functional openings and a BBB-like phenotype that is perfusable and facilitates the diffusion of oxygen (209, 210).

HIV is also thought to exacerbate age-associated cognitive decline. Cognitive deficits are twice as prevalent in PWH over 40 years of age than in their uninfected counterparts, and the risk of developing these deficiencies is disproportionately increased with age, of further importance in the current era, in which the HIV population is drifting progressively toward an older demographic (211–214). Aged human organoids could elucidate this collective effect of aging and HIV on the brain and associated neuropathological mechanisms that may occur. Previous work investigating the role of HIV infection in cellular aging and dysfunction can be easily implemented in a brain organoid model, such as assessing telomere length, toxic protein aggregate formation (i.e., amyloid-β and α-synuclein), and characterization of dysfunctional autophagy or senescence-activated secretory phenotype development (215–218). However, aged brain organoids require long-term maintenance and a stable and growth-arrested state. To overcome these issues, several studies attempted to “speed up the aging process” by obtaining cells from individuals affected with progeroid syndrome for iPSC generation or exposing brain organoids to reactive oxygen species, inflammation, or radiation to mimic stress-associated aging (219–222). Furthermore, as immature brain organoids can reflect the early processes of neurogenesis, the effect of HIV on early neurodevelopment could be evaluated, as vertical transmission persists (223, 224).

Although the use of brain organoid models to study neuroHIV is still in its infancy, rapid advancements highlight their utility in understanding mechanisms of neuropathology and viral persistence as well as testing of curative and neuropathological therapeutic approaches to ameliorate brain injury in PWH. Numerous HIV-focused research laboratories are actively pursuing methodological routes to determine the physiological relevance and predictive capability of brain organoids, and these ongoing efforts are expected to add value to current organoid technology to better explore the complexities of HIV within the CNS.
